# Lebetin 2, a Snake Venom-Derived Natriuretic Peptide, Attenuates Acute Myocardial Ischemic Injury through the Modulation of Mitochondrial Permeability Transition Pore at the Time of Reperfusion

**DOI:** 10.1371/journal.pone.0162632

**Published:** 2016-09-12

**Authors:** Bochra Tourki, Philippe Matéo, Jessica Morand, Mohamed Elayeb, Diane Godin-Ribuot, Naziha Marrakchi, Elise Belaidi, Erij Messadi

**Affiliations:** 1 Laboratoire des Venins et Biomolécules Thérapeutiques (LR11IPT08) et Plateforme de Physiologie et de Physiopathologie Cardiovasculaires (P2C), Institut Pasteur de Tunis, Université Tunis El Manar, Tunis, Tunisia; 2 Université Carthage Tunis, Bizerte, Tunisia; 3 Laboratoire de Signalisation et Physiopathologie Cardiovasculaire, UMR-S 1180, Faculté de Pharmacie, Université Paris Sud, Paris, France; 4 Laboratoire d’Hypoxie et Physiopathologie Cardiaque, Inserm U1042, Faculté de Pharmacie, Université Grenoble Alpes, Grenoble, France; Emory University, UNITED STATES

## Abstract

Cardiac ischemia is one of the leading causes of death worldwide. It is now well established that natriuretic peptides can attenuate the development of irreversible ischemic injury during myocardial infarction. Lebetin 2 (L2) is a new discovered peptide isolated from *Macrovipera lebetina* venom with structural similarity to B-type natriuretic peptide (BNP). Our objectives were to define the acute cardioprotective actions of L2 in isolated Langendorff-perfused rat hearts after regional or global ischemia-reperfusion (IR). We studied infarct size, left ventricular contractile recovery, survival protein kinases and mitochondrial permeability transition pore (mPTP) opening in injured myocardium. L2 dosage was determined by preliminary experiments at its ability to induce cyclic guanosine monophosphate (cGMP) release without changing hemodynamic effects in normoxic hearts. L2 was found to be as effective as BNP in reducing infarct size after the induction of either regional or global IR. Both peptides equally improved contractile recovery after regional IR, but only L2 increased coronary flow and reduced severe contractile dysfunction after global ischemia. Cardioprotection afforded by L2 was abolished after isatin or 5-hydroxydecanote pretreatment suggesting the involvement of natriuretic peptide receptors and mitochondrial K_ATP_ (mitoK_ATP_) channels in the L2-induced effects. L2 also increased survival protein expression in the reperfused myocardium as evidenced by phosphorylation of signaling pathways PKCε/ERK/GSK3β and PI3K/Akt/eNOS. IR induced mitochondrial pore opening, but this effect was markedly prevented by L2 treatment. These data show that L2 has strong cardioprotective effect in acute ischemia through stimulation of natriuretic peptide receptors. These beneficial effects are mediated, at least in part, by mitoK_ATP_ channel opening and downstream activated survival kinases, thus delaying mPTP opening and improving IR-induced mitochondrial dysfunction.

## Introduction

Myocardial infarction is one of the leading causes of death worldwide. Cardiac ischemia resulting from coronary occlusion leads to tissue hypoxia, cellular necrosis and apoptosis and organ dysfunction. Although reperfusion is the most straightforward treatment for limiting infarct size (IS), reperfusion has been shown to exacerbate myocardial damage in experimental and clinical settings [1]. This emphasizes need of finding new pharmacological agents capable of preventing ischemia-reperfusion (IR) injury. Recent works indicate that B-type natriuretic peptide (BNP) can attenuate irreversible ischemic injury in man [2], in vivo animal models [3,4] and in isolated hearts [5–7]. BNP has hypotensive, natriuretic and diuretic properties [8] and inhibits the sympathetic nervous system and the renin-angiotensin-aldosterone axis [9] leading to a decrease in pre- and after- load, thus maintaining blood supply to myocardial cells. BNP also has anti-proliferative and anti-fibrotic properties and thus may be involved in preventing cardiac remodelling [10].

Mechanisms involved in the effect of BNP in ischemia include activation of natriuretic peptide receptor type A (NPR-A) and stimulation of guanylyl cyclase (GC) to increase intracellular cyclic guanosine monophosphate (cGMP)-dependent protein kinase G (PKG) pathway [11] and subsequent triggering of mitochondrial K_ATP_ (mitoK_ATP_) channel opening [5,12]. However, growing evidence suggests that BNP could activate a cGMP-independent pathway by binding to Gi-protein coupled natriuretic peptide receptor type C (NPR-C) [13] and activating downstream PI3K/Akt/eNOS and MAPK/ERK pathways [5,6,14]. Mitochondria is recognized to have a critical role during myocardial IR in promoting both necrosis and apoptosis in association with opening of mitochondrial permeability transition pore (mPTP) and subsequent release of apoptotic signaling molecules. BNP has been suggested to mediate mPTP inhibition at the time of reperfusion [15], but this has not been confirmed in ischemic heart.

Snake have produced some of the more interesting natriuretic peptides [16] having greater potency and increased stability as compared to the human family members [16–19] and displaying similar activity in IR through a NPR-A/cGMP-mediated signaling [20,21]. Previous preclinical and clinical studies have shown that these venom-derived peptides can act on multiple disease processes that play a role in negative outcomes associated with cardiac ischemia [22]. Certain clinical results have demonstrated that venom-derived natriuretic peptides may represent a superior treatment solution by offering therapeutic benefits in chronic heart failure [23,24].

Lebetin 2 (L2) is a 4 kDa peptide isolated from *Macrovipera lebetina* venom [25]. This compound has structural homology to BNP [16,25]. In this study, we initially determined the optimal concentration of L2 to induce a sufficient increase in cGMP synthesis without producing hemodynamic effects in normoxic perfused rat hearts. We then examined the cardioprotective effects of L2 against acute IR injury in isolated rat hearts. We further assessed cellular and molecular mechanisms underlying the observed effects.

## Materials and Methods

### Animals

Male Wistar rats (280–300 g, Janvier Labs, l’Arbresle, France) were used for the study. They were housed in climate controlled conditions and had unrestricted access to standard rat chow and drinking water. This investigation was carried out in strict accordance with the recommendations in the Guide for the Care and Use of Laboratory Animals of the National Institutes of Health (NIH Pub. No. 85–23, Revised 1996). All experimental procedures were approved by the University Grenoble Alpes Animal Research Ethic Committee (authorization no. 184_UHTA_U1042_CA_03).

### Isolated heart preparation

Animals were anaesthetized by intraperitoneal injection of pentobarbital sodium (60 mg/kg). Hearts were rapidly excised and immediately immersed in 4°C Krebs-Henseleit (K-H) buffer solution (NaCl 118 mM, KCl 4.7 mM, CaCl_2_ 1.8 mM, MgSO_4_ 1.2 mM, KH_2_PO_4_ 1.2 mM, NaHCO_3_ 25.2 mM and glucose 11 mM). The aortic stumps were then cannulated and hearts were perfused retrogradely using the Langendorff technique at a constant pressure (75 mmHg) with gassed K-H buffer solution (95% O_2_−5% CO_2_). A water filled balloon (Hugo Sachs, n° 4, Les Ulis, France) coupled to a pressure transducer (ADInstruments, Paris, France) was inserted in the left ventricular cavity via the left atrium for pressure recording. Left ventricular end-diastolic pressure (LVEDP) was adjusted to 5–20 mmHg. Myocardial temperature was measured by a thermoprobe inserted into the left ventricle and was maintained constant close to 37°C.

### cGMP release and assessment of cardiac function in normoxic hearts

We determined the dose of L2 that would induce the maximum increase in coronary cGMP release without any effect on cardiac contractility. We compared these effects to those induced by BNP in the same conditions. Dose of BNP (10 nM) was chosen based on previous studies [5–7]. The equivalent dose of L2 was determined using increasing doses from 10 to 200 nM.

Thirty-two rats were assigned randomly to three groups. Rat hearts were stabilized for 20 min and then were perfused for 20 min with either vehicle (K-H buffer solution, control group, n = 8), BNP (10 nM, n = 14) or L2 (10, 100 or 200 nM, n = 10). The coronary effluent was collected 5 min after the start of the infusion and rapidly frozen at -80°C. cGMP concentration was determined by an enzyme immunoassay kit (cGMP Direct Immunoassay Kit, BioVision, CA, USA).

Cardiac hemodynamics: heart rate (HR), left ventricular developed pressure (LVDP), left ventricular end-diastolic pressure (LVEDP), maximum (dPmax/dt) and minimum (dPmin/dt) rate of rise of left ventricular pressure were recorded up to 50 min from the start of the infusion to assess cardiac function in normoxic conditions.

### Myocardial ischemia-reperfusion procedures

After 20 min stabilisation, rat hearts were subjected to either 30 min regional or global ischemia followed by 90 min reperfusion [26].

For regional IR, the left main coronary artery was occluded, 1 mm from the tip of the left atrium, with a 4–0 silk suture threaded through a plastic snare to permit reversible occlusion of the coronary artery. Reperfusion was achieved by loosening the suture.

For global IR, coronary occlusion was induced by clamping the aortic inflow lines and coronary flow was restored by removing the clamp.

**Fig 1:**
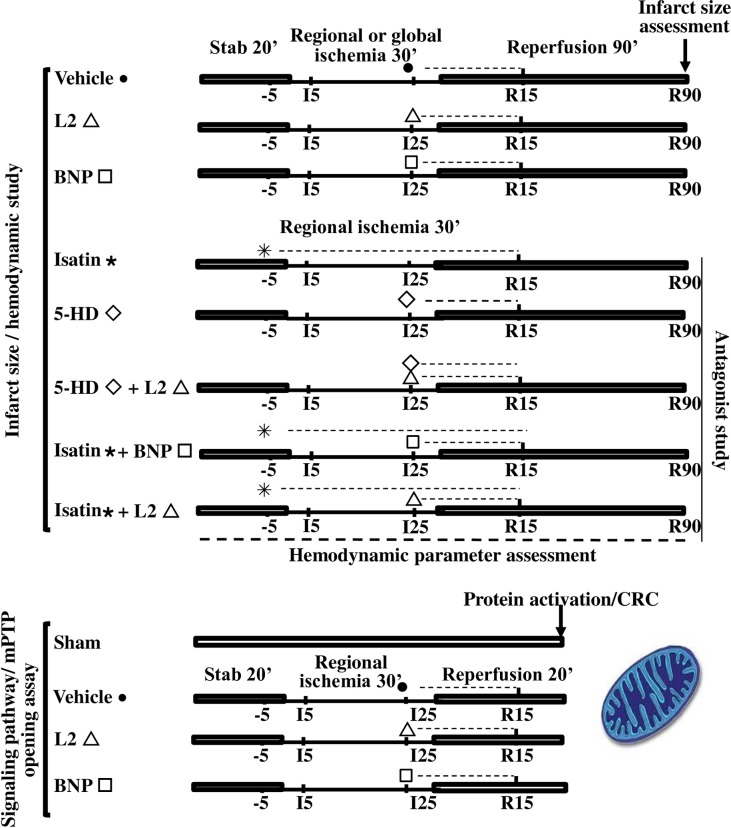
Ischemia-reperfusion experimental design. Dotted lines represent time course of natriuretic peptide and/or antagonist perfusion. Thin solid lines show regional or global ischemia and full solid lines show stabilisation and reperfusion periods. For infarct size and hemodynamic experiments, all hearts underwent 30 min of regional or global ischemia followed by 90 min reperfusion. L2 (200 nM) or BNP (10 nM) were administered at the time of reperfusion and drug perfusion was maintained for 20 min. For inhibitor study, the natriuretic peptide receptor antagonist isatin (100 μM) was perfused 5 min before regional ischemia until 20 min reperfusion. Mitochondrial ATP-sensitive potassium channel (mitoK_ATP_) blocker, 5 hydroxydecanoate (5-HD, 10 μM) was perfused 5 min before reperfusion for 20 min. For signaling pathway studies and mitochondrial permeability transition pore opening assay, hearts underwent either sham operation or 30 min of regional ischemia followed by 20 min reperfusion.

Three experimental groups with regional IR and three others with global IR (n = 9 to 10 per group) were studied (Fig 1). Hearts were stabilized for 20 min and then subjected to IR without treatment (control groups) or with L2 (200 nM) or BNP (10 nM) perfusion. Drug administration was started at the time of reperfusion and maintained for 20 min.

For mechanistic studies (Fig 1), five additional groups underwent regional IR (n = 6 to 8 per group). To study the involvement of natriuretic peptide receptors in L2 or BNP-induced effects, the natriuretic peptide receptor antagonist isatin (100 μM) was perfused 5 min before ischemia and maintained until 20 min reperfusion, alone or before L2 (200 nM) or BNP (10 nM) perfusion. To examine the role of mitoK_ATP_ channel opening, the selective mitoK_ATP_ channel blocker 5-hydroxydecanote (5-HD, 10 μM) was perfused 5 min before reperfusion and maintained 20 min, alone or in coperfusion with L2.

### Infarct size measurement

In regional IR experiments, at the end of reperfusion, the coronary artery was reoccluded. Evans blue dye (2%) was then injected through the cannulated aorta to delineate the area at risk (AR), which remained unstained by the Evans blue solution. The heart was removed and the left ventricle (LV) excised and frozen at -80°C for 15 min, and cut from apex to base into 2 mm-thick transverse slices (6 or 7 per heart). To delineate the IS, the slices were incubated at 37°C with buffered 1% 2,3,5-triphenyltetrazolium chloride (TTC) solution for 10 min, and were then fixed in a buffered 10% formalin solution for 24 h before being photographed. AR and IS were quantified by a blinded observer using a computerized planimetric technique (ImageJ software, NIH, Bethesda, Maryland, USA). IS was expressed as a percentage of AR.

In global IR experiments, the whole heart was removed rapidly at the end of reperfusion and left LV was cut into slices and directly incubated in TTC solution (1%) for 10 min as described above. IS was expressed as a percentage of LV.

### Post-ischemic cardiac function and coronary flow assessment

HR, LVDP, LVEDP, dPmax/dt and dPmin/dt were continuously recorded (8 channels, MacLab ADInstruments, Paris, France) and measured at the end of stabilisation period and at various regular times of ischemia and reperfusion. Values were averaged to assess cardiac function. Coronary flow (CF) was measured by timed collection of coronary effluent at regular intervals using a calibrated tube and expressed relative to heart weight (ml min^-1^g^-1^).

### Western blot analysis

To study the involvement of survival kinases in the observed cardioprotection, rat hearts underwent 30 min regional ischemia followed by 20 min reperfusion which was demonstrated to be sufficient to induce protein phosphorylation [27]. Four experimental groups were studied (n = 4 to 7 per group). One group was sham operated and the others underwent IR and received either K-H buffer (control group), L2 (200 nM) or BNP (10 nM), 5 min before reperfusion (Fig 1).

At the end of the experiment, the heart was excised and washed in cold physiological serum, dissected and the AR was rapidly frozen in liquid nitrogen to prevent protein de-phosphorylation. Myocardial tissue was later homogenized in 200 μl of lysis buffer [Tris-HCl 20 mM pH 7.5, NaCl 150 mM, EDTA 5 mM, Triton X100 1%, Tween 20 0.1%, with protease and phosphatase inhibitors (“Complete and PhosSTOP” Roche, Roche, Meylan, France)], and centrifuged 20 min at 13,000 rpm. The supernatant was ultracentrifuged at 100,000 g (1 h, 4°C) to collect the cytosolic fraction. Cytosolic proteins (50–150 μg) were separated on 10% SDS-polyacrylamide gels and transferred to PVDF membranes (Hybond P Sandwiches 0.45 PVDF, Amersham, GE Healthcare Europe GmbH, Saclay, France). Non specific proteins were then blocked by incubating membranes in Tris Buffer Saline-Tween 20 (0.05%) (T-TBS)-Bovine Serum Albumin (BSA, 5%), for 1 hour at room temperature. Phosphorylated (phospho-) Akt (1:500) and Akt (1:1000), phospho-PI3K (1:1000) and PI3K (1:1000), phospho-eNOS (1:500) and eNOS (1:500), phospho-ERK1/2 (1:1000) and ERK1/2 (1:2500), phospho-GSK3β (1:500) and GSK3β (1:500) (Cell Signaling, Danvers, MA, USA) and phospho-PKCε (1:1000) and PKCε (1:1000) antibodies (Santacruz, La Jolla, CA, USA) were then incubated in T-TBS-BSA (1%) or T-TBS-non fat dry milk powder (5%) overnight, at 4°C. After washing, bound antibody was visualized by use of horseradish peroxidase-conjugated goat anti-rabbit or anti-sheep antibody (1:5000, Cell Signalling, Saint Quentin, Yvelines, France) for 1 hour at room temperature. Loading and quality transfer were evaluated by tubulin staining (1:1000, Santa cruz, La Jolla, CA, USA). Enhanced chemiluminescence was performed with the ECL Western blot detection kit (Amersham, GE Healthcare Europe GmbH, saclay, France) according to the manufacturer’s instructions, and blots were exposed to BioRad Camera. Density of protein bands was computerized (ImageJ software, NIH, Bethesda, Maryland, USA). Data are normalized to tubulin (Sigma-Aldrich, Saint Quentin Fallavier, France) and expressed relative to sham group values.

### mPTP opening assay

Assessment of mPTP opening was performed on AR taken from the hearts previously used for signaling studies (Fig 1). Four experimental groups were studied: one group was sham operated and the others underwent regional IR and received either K-H buffer (control group), L2 (200 nM) or BNP (10 nM), 5 min before reperfusion. In a second set of experiments, L2, BNP or mPTP inhibitor cyclosporin A (CsA) were added at 1 μM to in vitro mitochondria isolated from hearts submitted beforehand to sham or IR operation.

Preparation of mitochondria was performed as previously described [28]. After 20 min reperfusion, hearts were excised while still beating and immediately placed in cold isolation buffer A (70 mM sucrose, 210 mM mannitol and 1 mM EDTA in 50 mM Tris-HCl, pH 7.4 at 4°C). Myocardial samples taken in the AR (300–550 mg) were used for mitochondria isolation. The tissue was finely minced with scissors and homogenized in the same buffer (1 ml buffer/0.1 g of tissue) using a manual Kontes and Potter-Elvehjem tissue grinders. The homogenate was centrifuged at 1,300 g for 3 min at 4°C. The pellet was discarded and the supernatant was filtered and centrifuged at 10,000 g for 10 min at 4°C. The mitochondrial pellet was then resuspended in 35 μl isolation buffer B (70 mM sucrose and 210 mM mannitol in 50 mM Tris-HCl, pH 7.4 at 4°C). Mitochondrial protein concentration was measured using the Bradford Method with BSA as standard [29].

Extramitochondrial Ca^2+^ fluorescence was measured with a Hitachi F2500 spectrofluorimeter in the presence of 0.5 μM Calcium Green-5N, with excitation and emission wavelengths set at 500 and 530 nm, respectively. Isolated mitochondria (260 μg of protein) were suspended in 2 ml of buffer C [150 mM sucrose, 50 mM KCl, 2 mM KH_2_PO_4_, and 5 mM succinic acid in 20 mM Tris/HCl (pH 7.4)]. Samples were pre-incubated for 90 sec in the cuvette, and 10-nmol CaCl_2_ pulses were applied every 60 sec in the spectrofluorometer. Each 10-nmol CaCl_2_ pulse causes a peak of extramitochondrial Ca^2+^ concentration that returned to near-baseline level when Ca^2+^ entered the mitochondria via the Ca^2+^ uniporter [30]. With increasing Ca^2+^ loading, the extramitochondrial Ca^2+^ concentration started accumulating, reflecting a lower capacity for mitochondria Ca^2+^ uptake, which was followed by a sustained Ca^2+^ increase indicating a massive release of the mitochondria Ca^2+^ by the mPTP opening, as previously described [31,32]. CRC was defined as the amount of Ca^2+^ required to trigger this massive Ca^2+^ release [28,33]. It is used here as an indicator of the mPTP sensitivity to Ca^2+^ and expressed as nanomoles of CaCl_2_ per milligram of mitochondrial proteins.

### Drugs and chemical reagents

Lebetin 2 (L2, 4 kDa) was isolated from venoms that were obtained from mature Tunisian snake *Macrovipera lebetina* housed in the serpentarium of the Institut Pasteur of Tunis. Venoms were stored at -20°C until use and L2 was then purified by preparative reversed phase medium-pressure liquid chromatography as previously described [25]. Briefly, a pool of venom was gel-filtered on a Sephadex G-75 column and then the L2 fraction purified by analytical reverse phase HPLC on a C8 column. Elution was performed using a linear gradient and monitored by measuring the absorbance (214 nm) with a Beckman 166 detector.

BNP (human recombinant peptide, 32 amino acids) and cyclosporin A (CsA) were purchased from Sigma-Aldrich (Saint Quentin Fallavier, France).

Isatin and 5-hydroxydecanoate (5-HD) were purchased from Cliniscience (Nanterre, France).

### Statistical analysis

Results are expressed as means ± S.E.M. Data were compared by two-way repeated measures analysis of variance (ANOVA) for cardiac hemodynamic parameters and one-way ANOVA for cGMP release, IS, protein phosphorylation and CRC. ANOVA was followed by Student’s t test for further evaluation of differences between two means.

Values of *p*<0.05 were considered to be statistically significant. Statistical studies were performed using the JMP software system (JMP; SAS Institute Inc., Cary, NC, USA).

## Results

### Effect of L2 in normoxic hearts

**Fig 2:**
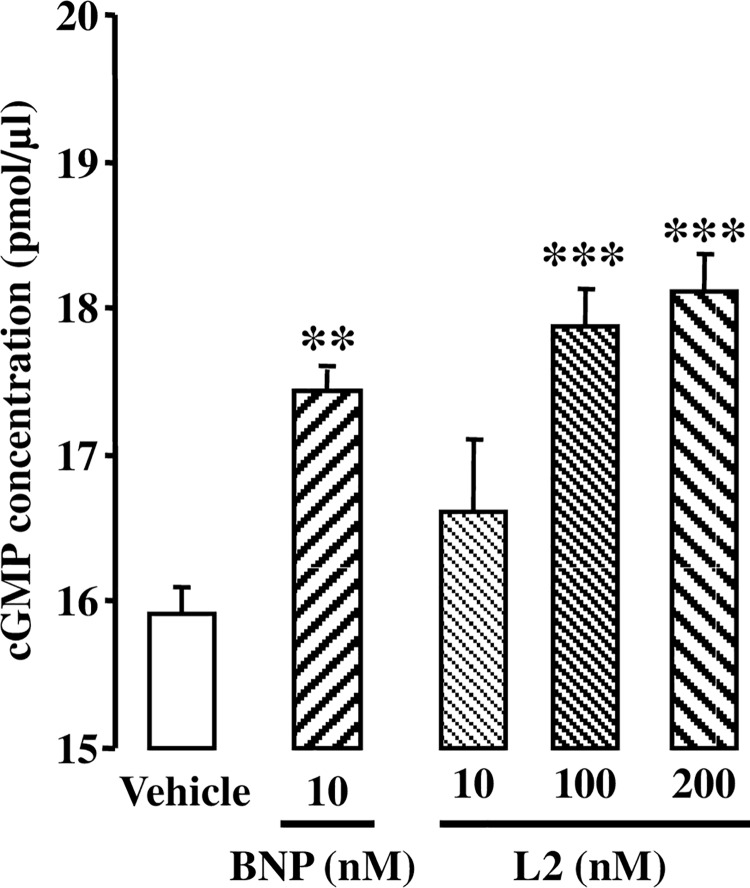
Effect of L2 on cGMP release. cGMP release was measured in the coronary effluent 5 min after BNP (10 nM) or L2 (10, 100 or 200 nM) perfusion under normoxic conditions. Data are mean ± SEM. n = 8–14 per group. **, *p*<0.01, ***, *p*<0.001 versus control (vehicle).

Changes in coronary cGMP release after BNP or L2 perfusion are shown in Fig 2. BNP (10 nM) significantly increased cGMP release in coronary effluent within 5 min compared to control group (17.4 ± 0.1 pmol/μl versus 15.9 ± 0.2 pmol/μl for control group, *p*<0.01). L2 also increased cGMP to the same extent at 100 and 200 nM (17.9 ± 0.3 pmol/μl and 18.1 ± 0.3 pmol/μl respectively, *p*<0.001 versus control group) but not at 10 nM.

During stabilisation period and in control vehicle-perfused rat hearts, mean baseline values for hemodynamic parameters were not statistically different among the experimental groups under normoxic conditions and these parameters remained unchanged after BNP (10 nM) or L2 (100 or 200 nM) perfusion (Table 1).

**Table 1:** Effect of L2 on cardiac contractility in normoxic hearts.

Normoxic study	n	HR	LVDP	LVEDP	dPmax/dt	dPmin/dt
		(bpm)	(mmHg)	(mmHg)	(mmHg/s)	(mmHg/s)
**Control study**	8					
**Stabilisation**		290 ± 9	116 ± 3.6	15.44 ± 2	3876 ± 42	-2356 ± 87
**Vehicle**		276 ± 12	109 ± 2.3	14.23 ± 3	3571 ± 76	-2120 ± 32
**BNP study**	14					
**Stabilisation**		288 ± 18	109 ± 5.6	16.28 ± 1	4053 ± 63	-2293 ± 106
**Vehicle**		271 ± 10	110 ± 3.7	15.84 ± 1	3673 ± 211	-2095 ± 97
**BNP (10 nM)**		277 ± 11	102 ± 3.3	15.03 ± 1	3425 ± 187	-2332 ± 105
**L2 study**	10					
**Stabilisation**		272 ± 13	112 ± 4	15.26 ± 2	4132 ± 21	-242 1 ± 87
**Vehicle**		266 ± 11	116 ± 2	15.47 ± 2	4087 ± 98	-2582 ± 78
**L2 (100 nM)**		287 ± 17	106 ± 5	14.74 ± 2	3958 ± 110	-2427 ± 107
**L2 (200 nM)**		291 ± 21	104 ± 7	15.55 ± 1	3993 ± 121	-2421 ± 92

Data are mean ± S.E.M. Heart rate (HR), left ventricular developed pressure (LVDP), left ventricular end-diastolic pressure (LVEDP), maximum (dPmax/dt) and minimum (dPmin/dt) rate of rise of ventricular pressure were recorded before and after L2 or BNP perfusion, at stabilisation period and 50 min after drug perfusion. bpm, beat per minute.

### Effect of L2 on myocardial post-ischemic injury

**Fig 3:**
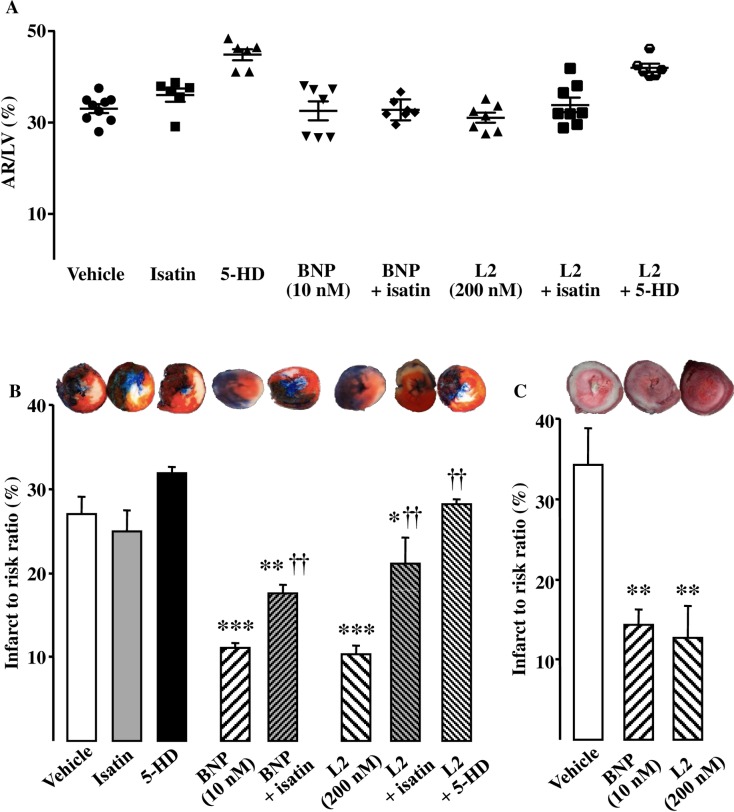
Effect of L2 on infarct size. (A) Area at risk ratio (%) in the experimental groups of hearts submitted to regional ischemia-reperfusion. (B) Infarct to risk ratio (%) in hearts submitted to regional ischemia-reperfusion after L2 or BNP perfusion alone or after pretreatment with the natriuretic peptide receptor antagonist isatin or the selective mitoK_ATP_ channel blocker 5-hydroxydecanote (5-HD). (C) Infarct to risk ratio (%) in hearts submitted to global ischemia-reperfusion after L2 or BNP perfusion. Myocardial infarction was induced by 30 min ischemia followed by 90 min reperfusion. Data are mean ± SEM. For number of animals see Table 2 and Table 3. *, *p*<0.05, **, *p*<0.01, ***, *p*<0.001 versus vehicle, ††, *p*<0.01 versus corresponding group not treated with antagonist.

The AR after ischemia were similar in all the experimental groups (Fig 3A). Under control conditions, IS averaged 27.0 ± 2.0% after regional IR and 34.7 ± 4.5% after global IR (Fig 3B and 3C). L2 (200 nM) reduced IS by approximately 60% after either regional or global IR [10.3 ± 1.0% (*p*<0.001) and 12.7 ± 3.9% (*p*<0.01) respectively; Fig 3B and 3C]. This IS-limiting effect was comparable to that obtained with BNP (10 nM) [11.1 ± 0.6% (*p*<0.001) and 14.5 ± 1.9% (*p*<0.01); Fig 3B and 3C].

**Fig 4:**
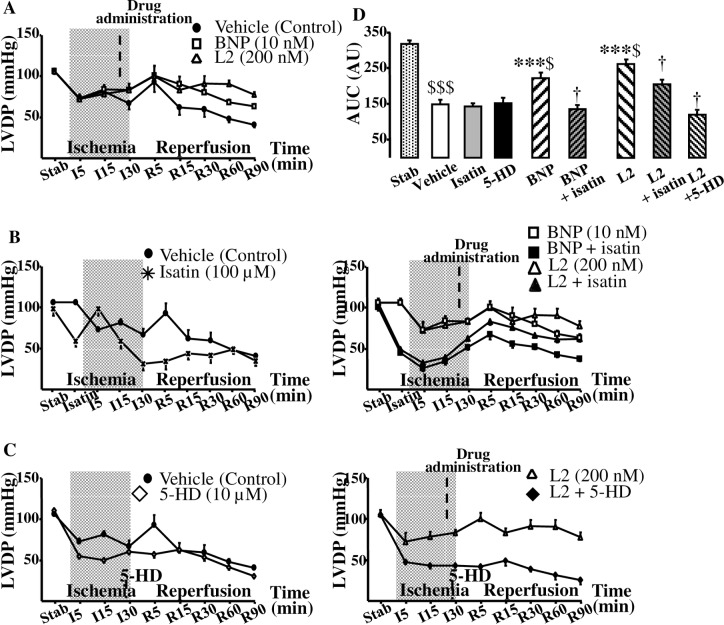
Effect of L2 on myocardial contractility in regional ischemia-reperfusion. (A) Effect of L2 on left ventricular developed pressure (LVDP, mmHg). (B) Effect of L2 on LVDP after natriuretic peptide receptor antagonist isatin pretreatment. (C) Effect of L2 on LVDP after mitoK_ATP_ channel blocker 5-hydroxydecanote (5-HD) treatment. (D) Area under curve (AUC, absolute variation of LVDP x time). Myocardial infarction was induced by 30 min regional ischemia followed by 90 min reperfusion. Data are mean ± SEM. For number of animals see Table 2. ***, *p*<0.001 versus vehicle, †, *p*<0.05 versus corresponding group not treated with antagonist, $, *p*<0.05, $ $ $, *p*<0.001 versus stabilisation values.

As was expected during post-ischemic reperfusion, the values of all functional parameters were decreased in all the groups compared to their respective baseline value (Fig 4A and 4D, Tables 2 and 3). Interestingly, L2 significantly improved all functional parameters after regional or global IR (Fig 4A and 4D, Tables 2 and 3) when BNP failed to improve the extremely severe contractile dysfunction of myocardium surviving global IR (Table 3). Coronary flow (CF), which was decreased after regional IR (Table 2), was restored after L2 treatment by 36% when remained unchanged in BNP-treated group.

**Table 2:** Effect of L2 on cardiac contractility in hearts subjected to regional ischemia-reperfusion experiments.

*Regional IR study*	n	HR		LVEDP		dPmax/dt		dPmin/dt		CF	
		(bpm)		(mmHg)		(mmHg/s)		(mmHg/s)		(ml/min)	
		Stab	R	Stab	R	Stab	R	Stab	R	Stab	R
**Control (vehicle)**	12	305 ± 8	255 ± 20$	18 ± 3	44 ± 8$	3469 ± 204	1182 ± 277$	-2873 ± 243	-1441 ± 38$	16.8 ± 0.7	8.5 ± 1.4$
**BNP (10 nM)**	10	310 ± 7	269 ± 15$	19 ± 1	33 ± 6$	3546 ± 143	2039 ± 231$*	-2950 ± 191	-1794 ± 136$	16.2 ± 0.8	9.3 ± 1.7$
**L2 (200 nM)**	10	305 ± 8	264 ± 13$	17 ± 5	28 ± 4$*	3417 ± 77	2450 ± 91$*	-2913 ± 151	-1899 ± 162$*	16.7 ± 0.8	11.5 ± 2.1$*
**Isatin (100 μM)**	7	304 ± 8	174 ± 16$*	21 ± 3	60 ± 21$	3462 ± 84	1054 ± 88$	-2858 ± 283	-1153 ± 89$	16.3 ± 0.6	7.3 ± 1.4$
**BNP + Isatin**	7	296 ± 9	244 ± 9$	21 ± 2	31 ± 9$	3248 ± 71	1306 ± 88$†	-2767 ± 66	-1120 ± 86$	16.7 ± 0.8	6.6 ± 1.0$†
**L2 + Isatin**	7	301 ± 10	189 ± 12$	20 ± 2	17 ± 4$*	3381 ± 61	1285 ± 118$†	-2929 ± 39	-1194 ± 144$	16.1 ± 0.5	6.7 ± 0.9$†
**5HD (10 μM)**	6	299 ± 10	106 ± 12$*	19 ± 3	49 ± 13$	3577 ± 100	1204 ± 50$	-2721 ± 78	-1221 ± 43$	16.9 ± 0.5	8.5 ± 1.3$
**L2 + 5-HD**	6	307 ± 7	165 ± 24$†	17 ± 4	33 ± 7$	3264 ± 82	877 ± 129$†	-2797 ± 82	-971 ± 67$†	16.3 ± 0.7	8.2 ± 0.7$†

Data are mean ± S.E.M. Heart rate (HR), left ventricular developed pressure (LVDP), left ventricular end-diastolic pressure (LVEDP), maximum (dPmax/dt) and minimum (dPmin/dt) rate of rise of ventricular pressure and coronary flow (CF) were recorded before and after L2 or BNP perfusion, at stabilisation period and at the end of reperfusion. The effect of the non selective natriuretic peptide receptor antagonist isatin and of the selective mitochondrial K_ATP_ channel blocker 5-hydroxydecanoate (5-HD) were also tested. bpm, beat per minute; IR, ischemia-reperfusion; Stab, stabilisation period; R, reperfusion.

*, *p*<0.05 versus corresponding vehicle value

^†^, *p*<0.05 versus corresponding group non treated with antagonist

^$^, *p*<0.05 versus corresponding stabilisation value.

**Table 3:** Effect of L2 on cardiac contractility in hearts subjected to global ischemia-reperfusion experiments.

*Global IR study*	n	HR		LVEDP		dPmax/dt		dPmin/dt		LVDP	
		(bpm)		(mmHg)		(mmHg/s)		(mmHg/s)		(mmHg)	
		Stab	R	Stab	R	Stab	R	Stab	R	Stab	R
**Control (vehicle)**	8	305 ± 8	252 ± 54	18 ± 1	141 ± 5$	3580 ± 128	544 ± 121	-2524 ± 92	-489 ± 66$	112 ± 7	23 ± 2$
**BNP (10 nM)**	8	310 ± 7	263 ± 53	18 ± 2	125 ± 7$	3580 ± 128	692 ± 120$	-2567 ± 94	-647 ± 101$	113 ± 2	31 ± 4$
**L2 (200 nM)**	9	305 ± 8	249 ± 21	20 ± 2	117 ± 10$*	3691 ± 94	828 ± 111$*	-2491 ± 62	-735 ± 72$*	113 ± 4	37 ± 3$*

Data are mean ± S.E.M. Heart rate (HR), left ventricular end-diastolic pressure (LVEDP), maximum (dPmax/dt) and minimum (dPmin/dt) rate of rise of ventricular pressure and left ventricular developed pressure (LVDP) were recorded before and after L2 or BNP perfusion, at stabilisation period and at the end of reperfusion. bpm, beat per minute; IR, ischemia-reperfusion; Stab, stabilisation period; R, reperfusion.

*, *p*<0.05 versus corresponding vehicle value

^$^, *p*<0.05 versus corresponding stabilisation value.

In inhibitory studies, the natriuretic peptide receptor antagonist isatin and the mitoK_ATP_ channel blocker 5-HD had no statistically significant effect on IS (Fig 3B) or myocardial contractility (Fig 4B, 4C and 4D, Table 2) when administered alone in regional IR. Natriuretic peptide receptor blockade by isatin suppressed the IS-limiting effect of L2 by 51% and BNP by 37% (Fig 3B), and significantly reduced functional recovery afforded by L2 and BNP (Fig 4B and 4D, Table 2).

Selective blockade of mitoK_ATP_ channels by 5-HD completely abolished the protection elicited by L2 on IS (Fig 3B) and contractile recovery (Fig 4C and 4D, Table 2).

### Effect of L2 on activation of cardioprotective signaling

**Fig 5:**
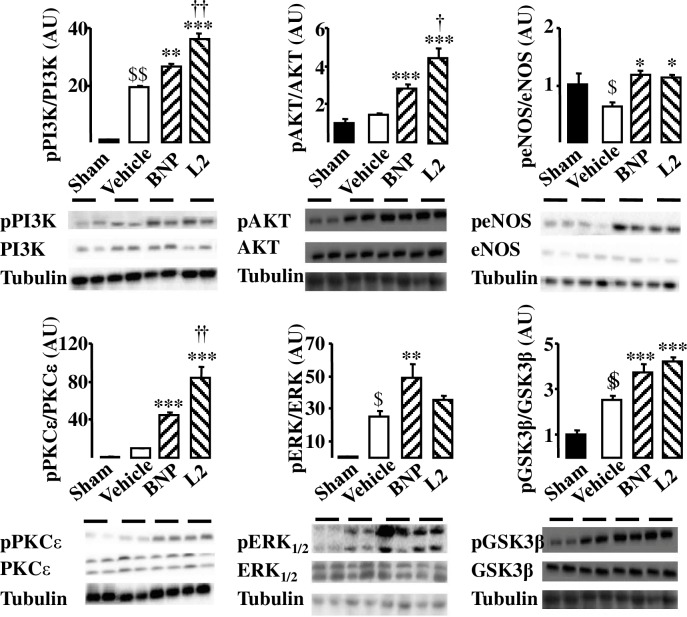
Effect of L2 on survival kinases in the ischemic myocardium. Protein phosphorylation was studied in sham operated hearts and in hearts subjected to 30 min regional ischemia followed by 20 min reperfusion and exposed to either vehicle (control), L2 (200 nM) or BNP (10 nM) perfusion. Data are presented as ratio of phosphorylated to total proteins and expressed relative to sham group values. Data are means ± SEM; AU: arbitrary unit; n = 4 per group. $, *p*<0.05, $ $, *p*<0.01 versus sham, *, *p*<0.05, **, *p*<0.01, ***, *p*<0.001 versus vehicle IR, †, *p*<0.05, ††, *p*<0.01, †††, *p*<0.001 versus BNP.

Having confirmed that L2 reduced myocardial ischemic injury, it was investigated whether this compound could generate any cardioprotective signal during IR. Since both NPR-A/cGMP/PKG-dependent [20,34] and NPR-C/G(i) [13,35] mechanisms trigger the major induced actions of natriuretic peptides, we have examined several components of these survival pathways which are known to be significantly affected by IR. To do that, phosphorylated PKCε/ERK/GSK3β and PI3K/Akt/eNOS expression was determined by Western blotting. In our experiments, IR did not induce any modification in phosphorylated Akt and PKCε expression (Fig 5). However, they were increased after L2 perfusion [3.00- and 8.88-fold increase respectively (*p*<0.001) compared with vehicle; Fig 5]. The amount of activated PI3K, ERK and GSK3β was increased after IR in the case of control hearts, while perfusion with L2 significantly enhanced the effect [pPI3K: 1.89- and pGSK3β: 1.92-fold increase (*p*<0.001) compared with vehicle; Fig 5], except for ERK where the increase was not significant. IR decreased the amount of activated eNOS and this was reversed when hearts were perfused with L2 [1.77-fold increase (*p*<0.05) compared with vehicle; Fig 5].

### Effect of L2 on mPTP opening

**Fig 6:**
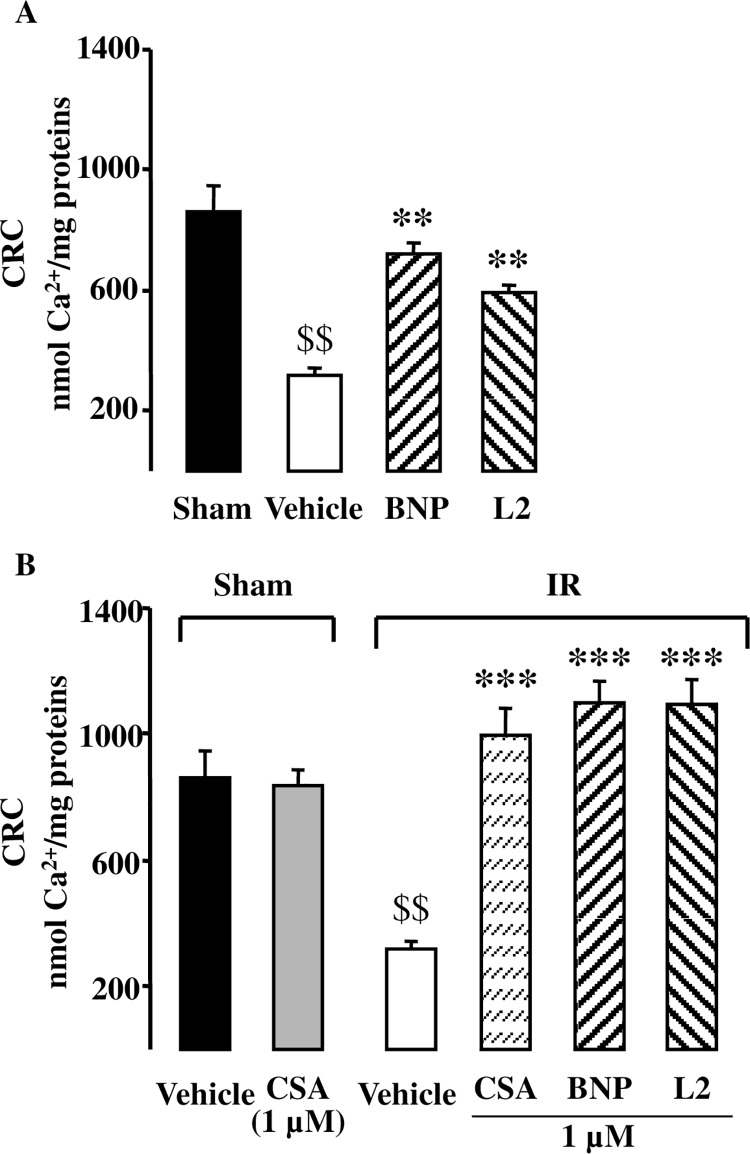
Effect of L2 on mitochondrial permeability transition pore opening. (A) Calcium retention capacity (CRC, nmol Ca^2+^/mg proteins) was studied in sham operated hearts and in hearts subjected to 30 min regional ischemia followed by 20 min reperfusion. (B) Effect of L2 (1 μM) and cyclosporin A (CsA, 1 μM) on CRC when added in vitro to isolated mitochondria that underwent sham or regional ischemia-reperfusion experiments. Data are mean ± SEM. n = 7 per group. $ $, *p*<0.01 versus sham group, **, *p*<0.01, ***, *p*<0.001 versus vehicle IR.

In order to determine if L2 could prevent the mPTP opening at the time of reperfusion, mitochondrial calcium retention capacity (CRC), used here as an indicator of the mPTP sensitivity, was measured. As expected, IR resulted in decrease of CRC by 63% compared to sham operated hearts (Fig 6A). L2 improved CRC after IR by 86% compared to vehicle treated group (Fig 6A). Consistent with these findings, we found that L2 at 1 μM, when added in vitro to ischemic mitochondria, increased CRC to the same extent as did CsA (1 μM) which is known to be the most specific inhibitor of the mPTP (Fig 6B).

### Discussion

The main findings of this study were that L2, a snake venom peptide with structural homology to BNP, exerts a cardioprotective effect by reducing IS and improving functional recovery in an ex vivo experimental model of IR injury.

Beneficial effects of BNP have been previously documented in chronic heart failure or in acute myocardial ischemia, either in clinical or experimental setting [4,5,7,36]. However, BNP has been reported to have a deleterious effect in certain cases [37,38] and its mechanism of action is not yet well established.

In the present study, we used dosages of BNP and L2 that we have documented in preliminary experiments to increase similarly cGMP release in coronary effluent without having any effect on cardiac function under normoxic conditions. We found that cGMP release was increased in coronary effluent within 5 min after 10 nM BNP and 100 or 200 nM L2 administration. This is consistent with previous studies that showed increase of either cellular cGMP content or coronary cGMP release during the first minutes following natriuretic peptide infusion [39,40]. In our study, the actual cellular cGMP concentrations were not measured. However, cGMP release was successfully used in previous studies to assess the effect of treatments stimulating cGMP synthesis [41]. Changes in cGMP release into the coronary effluent have also been shown to be a reliable index of changes in myocardial cGMP synthesis [42].

Because 200 nM L2 induced a significant increase in cGMP release into the coronary effluent without any effect on cardiac function, we used this dose for IR studies. In the present work, we used two models of myocardial ischemia namely regional IR and global IR. Because in man myocardial ischemia is most commonly regional in nature [43] and widely studied in intact animals, we first studied regional IR in the Langendorff-perfused rat heart model to study the effect of L2 on infarct size and post-ischemic left ventricular dysfunction. However, although regional IR model corresponds to the clinical scenario, this technique is not a reliable model to differentiate changes in contractile dysfunction between protected and non protected hearts since necrotic area is not large enough to significantly affect cardiac work [44]. Therefore, L2 was tested in a model of global ischemia to confirm the observed effects. The model of global myocardial ischemia presents clear-cut disadvantages with respect to its relevance with acute coronary occlusion in patients [45], thus the use of regional and global IR could be a complementary approach to enable comprehensive assessment of post-ischemic left ventricular dysfunction.

Our results showed that after ischemia all AR were similar suggesting that there were no anatomical differences in the coronary vessels or left ventricles among hearts that could have influenced the results of IR. L2 reduced IS by approximately 60% after either regional or global IR and this effect was comparable to that obtained with BNP. Interestingly, the venom peptide improved post-ischemic contractile function either after regional or global ischemia, when BNP failed to improve the extremely severe contractile dysfunction of myocardium surviving global IR. L2 also elicited a marked increase in coronary flow by 36% when this parameter remained unchanged after BNP treatment as reported by Burley and coworkers [5,7]. Although L2 was found to be active in both regional and global IR, however, cardioprotection showed different trend between these two models as post-ischemic contractility was improved to a lesser extent in global IR compared to regional maneuver. This could be due to the more deleterious effect of global IR [46], thus L2 is less effective under such conditions to better restore contractile recovery.

Cardiac effects of L2 are mediated by natriuretic peptide receptors since L2 resulted in cGMP release in a dose-related manner and beneficial effects of L2 were largely reduced after natriuretic peptide receptor antagonist isatin pretreatment. This is consistent with previous observations of structural homology between L2 and BNP [16,25]. Myocardial natriuretic peptides are produced by isolated rat heart during IR [7,47]. However, whereas isatin did not induce any effect under basal conditions (in the absence of L2/BNP treatment), it reduced beneficial effects of L2 and exogenous BNP, suggesting that the level of endogenous natriuretic peptide production in that context was too low to provide cardioprotection [5,7]. Although high dose of isatin [5] was chosen in order to ensure efficient antagonism of L2 and BNP during IR, our data showed an incomplete blockade of isatin for L2 and BNP-mediated cardioprotection. As isatin preferentially blocks NPR-A and NPR-C [48], the observed cardioprotection by BNP and L2 under isatin may be due to their binding to NPR-B receptors [49,50]. These receptors are a structurally similar GC receptors and exist in the heart but at a lower abundance than NPR-A [51]. They are mainly activated by C-type natriuretic peptide (CNP) and could also be stimulated to a lesser extent by BNP leading to the same action than NPR-A binding [49]. The low abundance of this receptor in heart under basal conditions [51] may provide an explanation for why it has not been involved in the BNP and L2 action in the absence of treatment with isatin. NPR-A activation is the predominant mechanism mediating BNP actions [52], but alternatively, NPR-B could represent a compensatory mechanism of cardiprotection in the case of loss of the dominant receptor function. Recent evidence suggests that NPR-B could be involved in cardioprotection in rat ventricular cardiomyocytes [50] but their role is minor than that exerted by the NPR-A [53]. Furthermore, it has been recently reported that NPR-A down regulation is positively associated with an increased expression of NPR-B protein in rat hearts [51].

Overall, our results are consistent with previous studies showing that BNP reduces IS [4–7] and improves post-ischemic recovery [54]. As reported for BNP, the beneficial effect of L2 in acute cardiac ischemia can be related to inhibition of apoptotis [3], inflammation [55] and oxidative stress [56]. Furthermore, IR has been found to markedly decrease myocardial cGMP content in ischemic heart [42]. The increase in the release rate of cGMP following L2 administration and lack of cardioprotection in the presence of natriuretic peptide receptor antagonist, indicate that the protective effects of L2 against IR injury are at least due to normalization of cGMP content and its direct effect on reperfused cardiac myocytes. Restoration of normal cGMP contents by natriuretic peptides has been shown to improve Ca^2+^ kinetics, to inhibit platelet aggregation, to promote vasorelaxation [57] and to protect endothelial function [58] in reperfused myocardium.

Since both NPR-A/cGMP/PKG-dependent [20,34] and NPR-C/G(i) [13] mechanisms trigger the major induced actions of natriuretic peptides, we have investigated if components of these survival pathways were involved in the L2-induced effects. We found that L2 enhanced phosphorylated PKCε/ERK/GSK3β and PI3K/Akt/eNOS expression suggesting that both cGMP/PKG-dependent and independent pathways are involved in the L2-induced effects during IR. This is in agreement with recent data reporting that protective action of BNP in IR could involve PI3K/Akt/eNOS and PKCε/GSK3β pathways [5,6,14]. This further documents the similarity of action between L2 and BNP. For BNP, we showed for the first time that phosphorylated ERK1/2 expression was increased in reperfused myocardium while previous studies reported conflicting results concerning the interaction of BNP and ERK in the setting of cardiac ischemia [59,60]. Furthermore, venom peptide L2 was found to be more potent than BNP at increasing Akt, PI3K and PKCε expression in reperfused myocardium, but failed to significantly increase ERK phosphorylation. Since, ERK activation is closely related to PKCε increase [61], we have expected much more increase of ERK in L2-treated hearts than in BNP group. Surprisingly, ERK was down regulated in L2-treated group. This could be due to overstimulation of PKCε which may induce MAPK phospahatase-1 (MKP-1) and thus causes down regulation of ERK [62].

Consistent with previous work [14], we found that IR decreased the amount of activated eNOS and this was reversed when hearts were perfused with L2. Recent data using NO synthase inhibitors suggest that NO contributes to the cardioprotection afforded by BNP [14]. Since myocardial IR injury is associated with an altered nitric oxide (NO)-dependent relaxation of conduit coronary arteries [63], it appears from the present study that NO signaling may constitute an important injury-limiting mechanism of L2 in ischemic myocardium. Release of NO restores endothelium-dependent vasodilation [64] and inhibits platelet aggregation and neutrophils adhesion [65], thus maintaining blood supply to injured myocardial cells.

Mitochondrial dysfunction plays a causal role in apoptosis and myocardial cell death during cardiac IR. The detrimental role of mitochondria in cardiac ischemia is related to the opening of mPTP at the time of reperfusion and the subsequent release of apoptotic proteins. The effect of BNP on mitochondrial function and mPTP opening in IR is not well defined. Recent studies showed that BNP protects ischemic myocardium by opening of mitoK_ATP_ channels which in turn preserves mitochondrial inner membrane permeability and mPTP integrity during IR [66]. BNP also attenuates mitochondrial dysfunction in cultured cardiomyocytes subjected to reoxygenation [15] but this effect has not been confirmed in ischemic hearts. To the best of our knowledge, our study is the first to show that protective effect of L2 as well as BNP against IR injury is associated with an improvement of mitochondrial CRC in reperfused myocardium, suggesting an inhibition of mPTP opening at the time of reperfusion. As demonstrated in the present study, L2 and BNP increased phosphorylated Akt, PKCε, ERK and GSK3β expression. Activation of Akt, PKCε, and ERK is known to be involved in limitation of lethal reperfusion injuries by preventing mitochondria permeability transition through phosphorylation of GSK3β [15,67]. PI3K/Akt/NO pathway, which we have shown to be increased after L2 treatment, has also been demonstrated to be involved in the inhibition of mPTP opening at the time of reperfusion thus decreasing apoptosis, as well as reducing IS and improving cardiac function after IR [68,69]. Recent data performed on isolated hearts suggest that mitoK_ATP_ channel opening contributes to the cardioprotection afforded by BNP by preserving mPTP integrity during IR [5,7,66]. In our experiments, protection elicited by L2 on IS and contractile recovery was reversed after mitoK_ATP_ channel blocker 5-HD pretreatment. These results argue for a role of mitoK_ATP_ channels as a relevant factor in the limitation of necrosis and apoptosis in the ischemic-reperfused rat heart. These observations are further confirmed by in vitro CRC experiments that demonstrated that L2 was as potent as CsA in conferring high resistance against Ca^2+^ loading to ischemic mitochondria. This is consistent with studies showing that inhibition of mPTP opening by CsA decreases IS and improves functional recovery after IR injury [70].

The present study shows that L2 when administered at the time of reperfusion, efficiently reduced the extent of myocardial injury by reducing IS and improving functional post-ischemic recovery. The data also suggest that L2 utilizes identical mechanism for cardioprotection as BNP does by activating natriuretic peptide receptors and mitoK_ATP_ channels, and subsequently delaying mPTP opening at the time of reperfusion, thus attenuating IR-induced damage. However, L2 has been found to be more potent than BNP in increasing coronary flow and improving contractile dysfunction of myocardium surviving global IR. Thus, more mechanistic investigation is required to further explore how L2 exerts this additional action. Our results, together with those reported previously with other snake venom-drived natriuretic peptides, provide conclusive evidence that L2, could be a strong candidate for the treatment of acute myocardial ischemia. Many of the reptilian peptides possess greater stability and less adverse side effects that give them advantages over their mammalian counterparts for therapeutic use. Recently, the chimeric peptide Cenderitide, designed from the snake venom dendroaspis natriuretic peptide (DNP) [71], has been found to exert potent cardioprotective action and is being evaluated in a phase II clinical trial for the treatment of cardiac ischemia [23,24]. Furthermore, unlike BNP, L2 presents the advantage to have a potent antiplatelet activity [72], which makes it a drug of choice for the treatment of acute myocardial infarction. However, L2 is a peptide isolated from a snake venom. The whole peptide may be immunogenic when administered repeatedly. Currently, it is possible to have a L2 synthetic analogue since small molecules pose fewer problems for design and chemical synthesis [73]. Furthermore, L2 subunit sequences are known and a recombinant version of L2 has already been produced in prokaryotic cells using *E*. *coli* and tested to determine if exhibits similar *in vitro* properties (Jed Jebali, personal communication, january 2016). L2 sequence could be adapted (to produce L2 derivates with less immunogenic response) for an appropriate use in clinical situation.

## Supporting Information

S1 Fig Effect of L2 on phosphorylated PI3K expression in the ischemic myocardium. Phosphorylation of PI3K was studied after 30 min regional ischemia followed by 20 min reperfusion, in sham operated hearts and in hearts subjected to ischemia-reperfusion and exposed to either vehicle (control), L2 (200 nM) or BNP (10 nM) perfusion, starting 5 min before reperfusion and maintained for 20 min. Tubulin was used as a house keeping protein control.
(TIFF)

S2 Fig Effect of L2 on phosphorylated Akt expression in the ischemic myocardium. Phosphorylation of Akt was studied after 30 min regional ischemia followed by 20 min reperfusion, in sham operated hearts and in hearts subjected to ischemia-reperfusion and exposed to either vehicle (control), L2 (200 nM) or BNP (10 nM) perfusion, starting 5 min before reperfusion and maintained for 20 min. Tubulin was used as a house keeping protein control.
(TIFF)

S3 Fig Effect of L2 on phosphorylated eNOS expression in the ischemic myocardium. Phosphorylation of eNOS was studied after 30 min regional ischemia followed by 20 min reperfusion, in sham operated hearts and in hearts subjected to ischemia-reperfusion and exposed to either vehicle (control), L2 (200 nM) or BNP (10 nM) perfusion, starting 5 min before reperfusion and maintained for 20 min. Tubulin was used as a house keeping protein control.
(TIFF)

S4 Fig Effect of L2 on phosphorylated PKCε expression in the ischemic myocardium. Phosphorylation of PKCε was studied after 30 min regional ischemia followed by 20 min reperfusion, in sham operated hearts and in hearts subjected to ischemia-reperfusion and exposed to either vehicle (control), L2 (200 nM) or BNP (10 nM) perfusion, starting 5 min before reperfusion and maintained for 20 min. Tubulin was used as a house keeping protein control.
(TIFF)

S5 Fig Effect of L2 on phosphorylated ERK1/2 expression in the ischemic myocardium. Phosphorylation of ERK1/2 was studied after 30 min regional ischemia followed by 20 min reperfusion, in sham operated hearts and in hearts subjected to ischemia-reperfusion and exposed to either vehicle (control), L2 (200 nM) or BNP (10 nM) perfusion, starting 5 min before reperfusion and maintained for 20 min. Tubulin served as molecular weight marker.
(TIFF)

S6 Fig Effect of L2 on phosphorylated GSK3β expression in the ischemic myocardium. Phosphorylation of GSK3β was studied after 30 min regional ischemia followed by 20 min reperfusion, in sham operated hearts and in hearts subjected to ischemia-reperfusion and exposed to either vehicle (control), L2 (200 nM) or BNP (10 nM) perfusion, starting 5 min before reperfusion and maintained for 20 min. Tubulin was used as a house keeping protein control.
(TIFF)
