# Supplementary figures and images for: Lebetin 2, a Snake Venom-Derived Natriuretic Peptide, Attenuates Acute Myocardial Ischemic Injury through the Modulation of Mitochondrial Permeability Transition Pore at the Time of Reperfusion
Source: PLoS One. 2016 Sep 12;11(9):e0162632. doi: 10.1371/journal.pone.0162632 (PMC5019389; doi:10.1371/journal.pone.0162632)

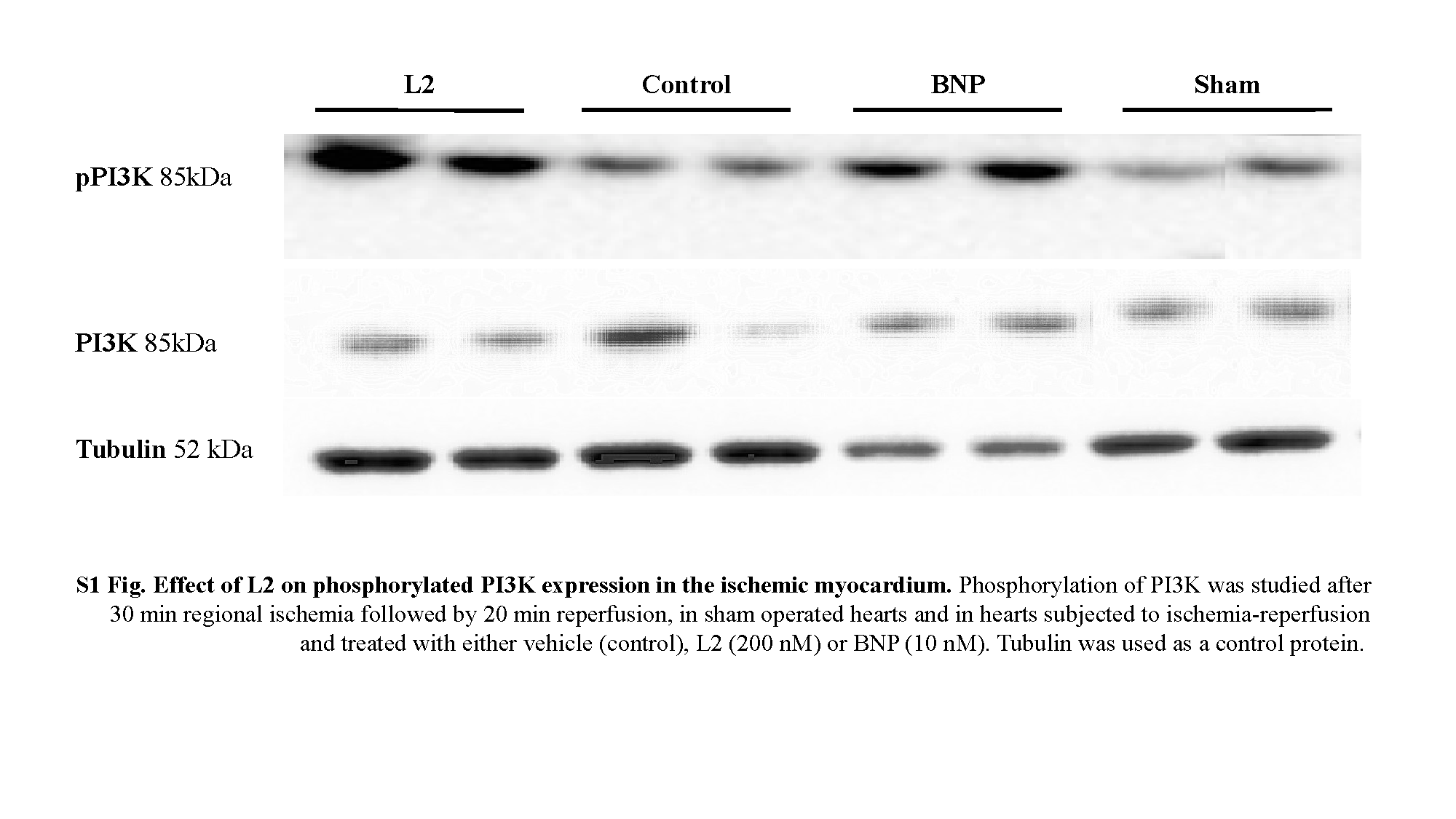

Supplement: S1 Fig — Phosphorylation of PI3K was studied after 30 min regional ischemia followed by 20 min reperfusion, in sham operated hearts and in hearts subjected to ischemia-reperfusion and exposed to either vehicle (control), L2 (200 nM) or BNP (10 nM) perfusion, starting 5 min before reperfusion and maintained for 20 min. Tubulin was used as a house keeping protein control. (TIFF) [file pone.0162632.s001.tiff]

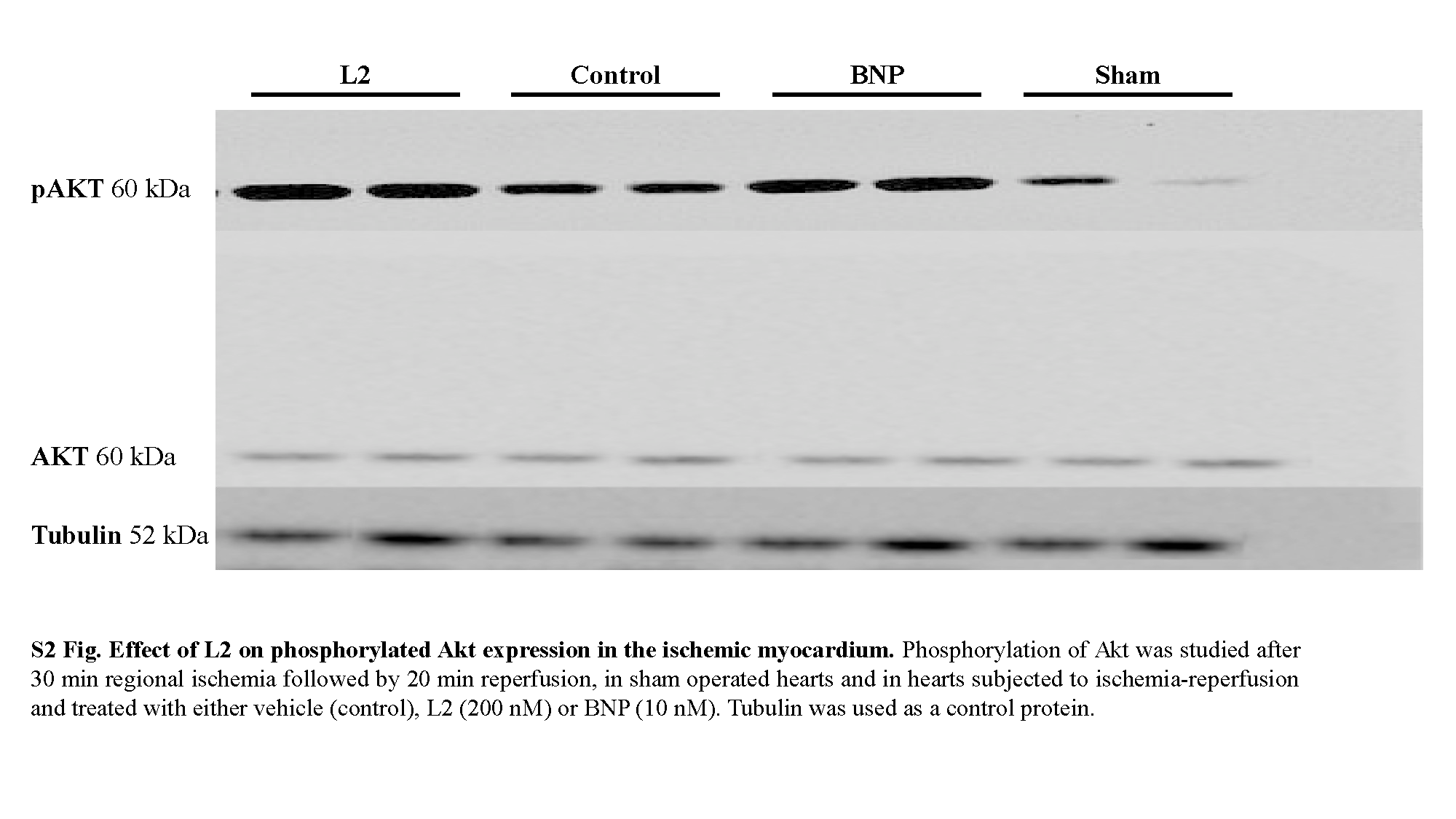

Supplement: S2 Fig — Phosphorylation of Akt was studied after 30 min regional ischemia followed by 20 min reperfusion, in sham operated hearts and in hearts subjected to ischemia-reperfusion and exposed to either vehicle (control), L2 (200 nM) or BNP (10 nM) perfusion, starting 5 min before reperfusion and maintained for 20 min. Tubulin was used as a house keeping protein control. (TIFF) [file pone.0162632.s002.tiff]

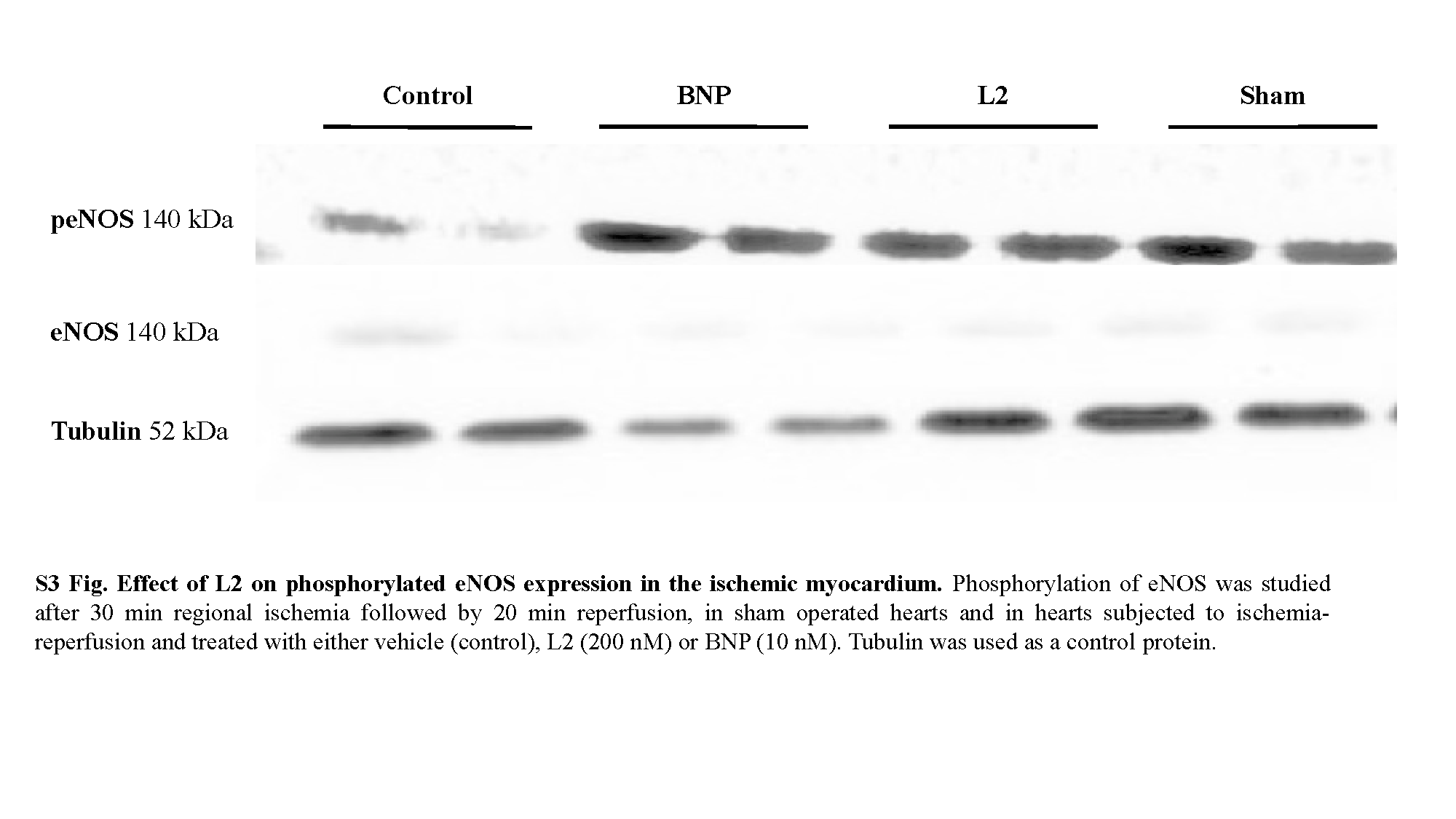

Supplement: S3 Fig — Phosphorylation of eNOS was studied after 30 min regional ischemia followed by 20 min reperfusion, in sham operated hearts and in hearts subjected to ischemia-reperfusion and exposed to either vehicle (control), L2 (200 nM) or BNP (10 nM) perfusion, starting 5 min before reperfusion and maintained for 20 min. Tubulin was used as a house keeping protein control. (TIFF) [file pone.0162632.s003.tiff]

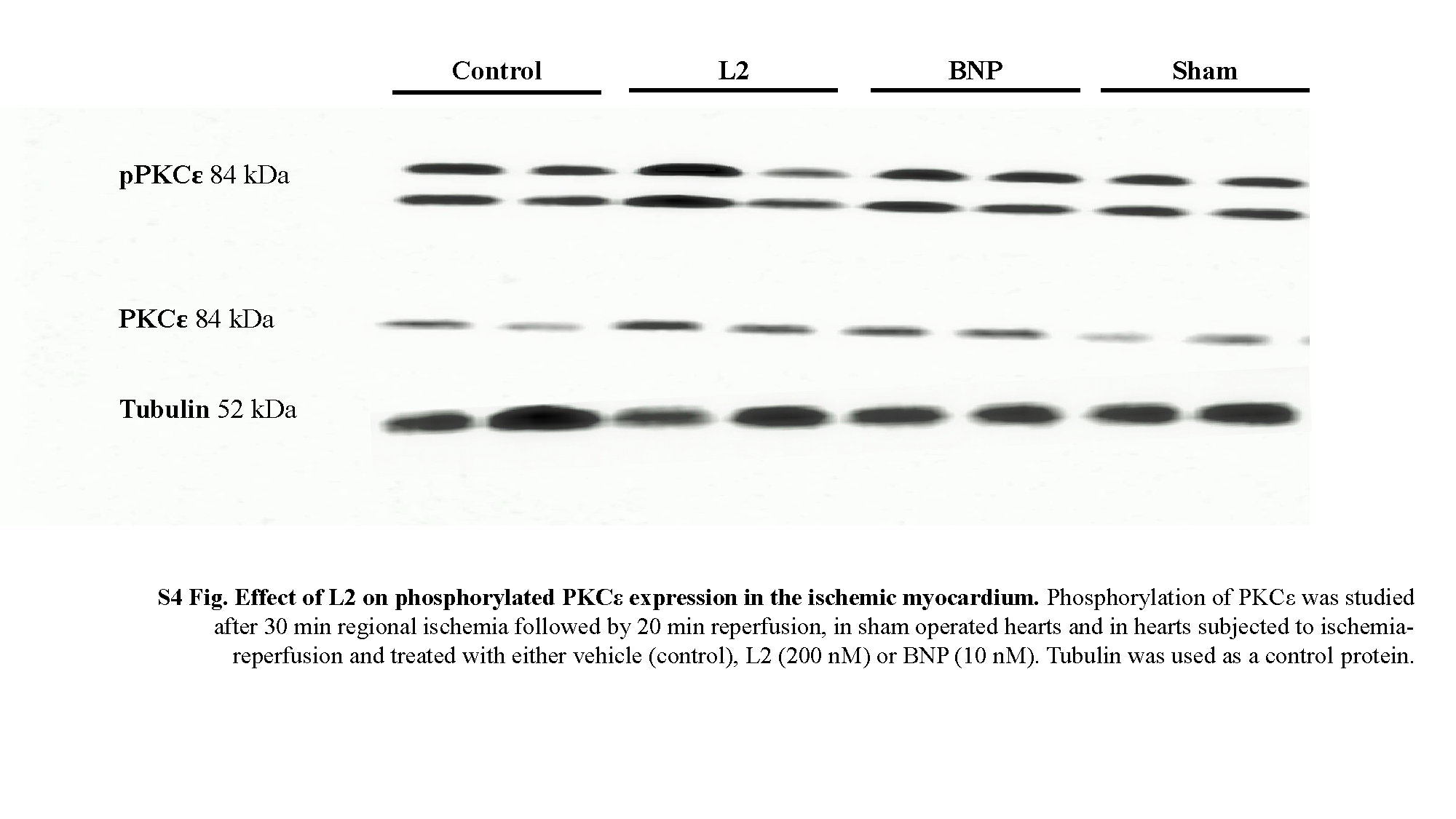

Supplement: S4 Fig — Phosphorylation of PKCε was studied after 30 min regional ischemia followed by 20 min reperfusion, in sham operated hearts and in hearts subjected to ischemia-reperfusion and exposed to either vehicle (control), L2 (200 nM) or BNP (10 nM) perfusion, starting 5 min before reperfusion and maintained for 20 min. Tubulin was used as a house keeping protein control. (TIFF) [file pone.0162632.s004.tiff]

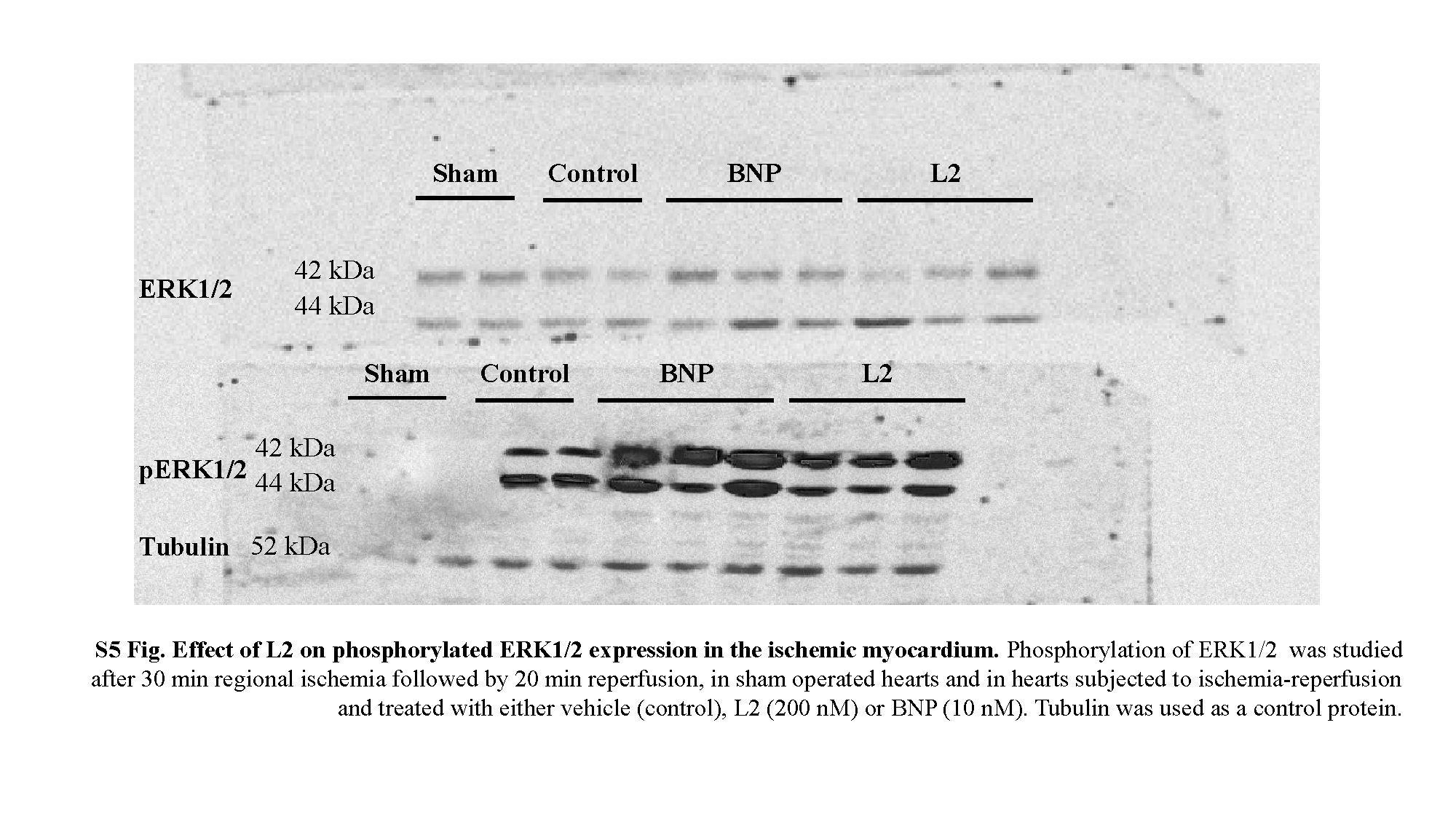

Supplement: S5 Fig — Phosphorylation of ERK1/2 was studied after 30 min regional ischemia followed by 20 min reperfusion, in sham operated hearts and in hearts subjected to ischemia-reperfusion and exposed to either vehicle (control), L2 (200 nM) or BNP (10 nM) perfusion, starting 5 min before reperfusion and maintained for 20 min. Tubulin served as molecular weight marker. (TIFF) [file pone.0162632.s005.tiff]

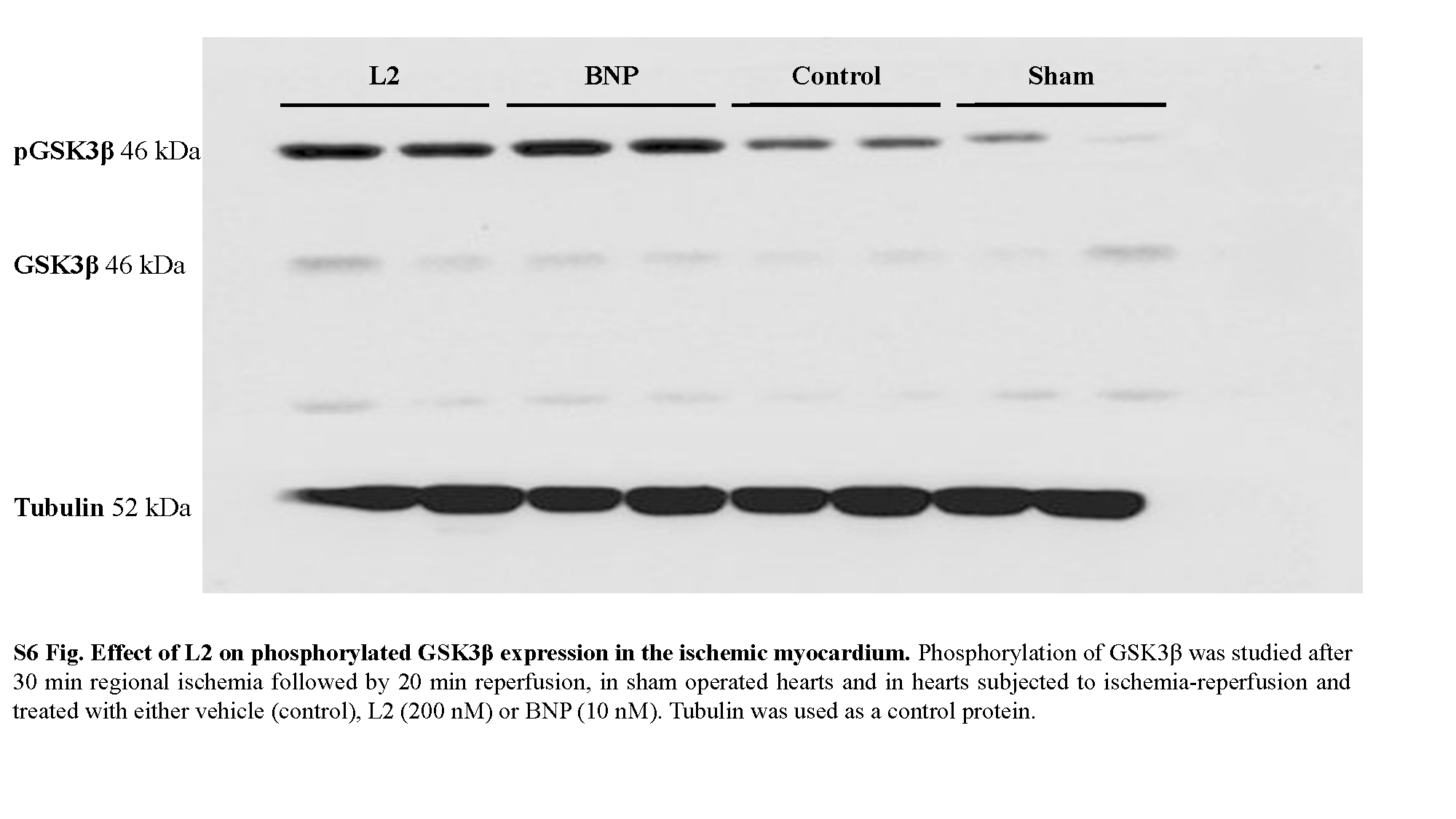

Supplement: S6 Fig — Phosphorylation of GSK3β was studied after 30 min regional ischemia followed by 20 min reperfusion, in sham operated hearts and in hearts subjected to ischemia-reperfusion and exposed to either vehicle (control), L2 (200 nM) or BNP (10 nM) perfusion, starting 5 min before reperfusion and maintained for 20 min. Tubulin was used as a house keeping protein control. (TIFF) [file pone.0162632.s006.tiff]
